# Exploring Quantum-Inspired Encoding Strategies in Neuromorphic Systems for Affective State Recognition

**DOI:** 10.3390/s26020568

**Published:** 2026-01-14

**Authors:** Fang Wang, Xiaoqiang Liang, Xingqian Du

**Affiliations:** 1 School of Optoelectronic Engineering, Xi’an Technological University, Xi’an 710021, China; wangfang86@xatu.edu.cn; 2 China North Vehicle Research Institute, Beijing 100072, China; 3 China Academy of Space Technology (Xi’an), Xi’an 710100, China; duxingqian16@mails.ucas.edu.cn

**Keywords:** affective computing, emotion recognition, adjacent feature entanglement, spiking encoding

## Abstract

In this paper, we explore the spiking encoding methodology within spiking neural networks for affective state recognition, deriving inspiration from the principles of quantum entanglement. A pioneering encoding strategy is proposed based on the strategic utilization of the quantum mechanical phenomenon of entanglement. By integrating quantum mechanisms into the spike-encoding pipeline, we aim to match the accuracy of existing encoders on emotion-classification tasks while retaining the inherently low-power advantage of spiking neural networks. Notably, leveraging the superposition of quantum bits and their potential quantum entanglement of adjacent values in feature space during encoding calculations, this quantum-inspired encoding paradigm holds substantial promise for augmenting information processing capabilities in brain-like neural networks. Through quantum observation, we derive spike trains characterized by quantum states, thereby establishing a foundation for experimental validation and subsequent investigative pursuits. We conducted experiments on emotion recognition and validated the effectiveness of our method.

## 1. Introduction

In light of the proliferation of large models across diverse sectors, the realm of Artificial Intelligence (AI) has ushered in a new epoch of advancement [1,2,3,4]. However, the substantial energy consumption associated with these large models constitutes a formidable concern that necessitates careful consideration [5,6,7,8]. This concern not only demands considerable financial investment but also gives rise to significant environmental ramifications. Consequently, scholars and industry stakeholders are actively seeking energy-efficient solutions [9]. Although intelligent applications such as face recognition and object detection have been deployed on a large scale, the focus of artificial intelligence is shifting from meeting utilitarian needs to addressing the more nuanced psychological needs of individuals, and even toward achieving more human-like decision-making mechanisms based on empathy [10,11]. In this context, the domain of affective computing emerges as a pertinent field, catering to higher-level requirements that manifest once fundamental daily needs are satisfied [12]. Consequently, there is a burgeoning interest in research pertaining to affective computing through the utilization of Spiking Neural Networks (SNNs) [13,14,15], representing a highly promising and interdisciplinary avenue of inquiry.

SNNs, denoted as third-generation neural networks [16], represent a computational mechanism inspired by the structure of the brain, finding increasing application across diverse domains. These networks have garnered notable attention in prominent conferences in recent times, owing to their distinctive merits, notably, reduced energy consumption and asynchronous operation [17]. These attributes are facilitated by a non-von Neumann hardware architecture characterized by a decentralized structure. This architectural framework permits asynchronous computing, surmounting the memory bottleneck inherent in the von Neumann architecture’s segregation of memory and computing functions. The amalgamation of computing and storage within a singular computing core, as exemplified in brain-like chips [9], presents a potential solution with low-energy implications, effectively mitigating the challenges associated with energy consumption in data handling processes.

The application of SNNs typically involves a sequential execution of distinct steps, encompassing spiking encoding, spiking neuron selection, SNN topology design, SNN training, and SNN inference [18]. Spiking encoding assumes a central role in data processing, given the intrinsic event-driven characteristics of SNNs, facilitating a natural adaptation to sequence data composed of binary elements (0/1). The fundamental operation of spiking encoding involves the transformation of conventional raw data, such as visual or auditory waveform data, into spiking trains characterized by binary states (0/1) via the implementation of a specified encoding methodology [18].

The existing spiking encoding methodologies predominantly manifest as two discernible types: rate encoding and temporal encoding [18]. Temporal encoding proficiently preserves the temporal attributes inherent in the initial floating-point data through its conversion into a spike emitted at fixed intervals within a predetermined temporal window. However, the application of temporal encoding is circumscribed due to its suboptimal performance in subsequent tasks. Temporal encoding is distinguished by its ability to ensure the sparsity of encoding results, a fundamental prerequisite for achieving low energy consumption during subsequent model inference. Another encoding technique is rate encoding, wherein the original floating-point data is encoded into a spike train emitted at a uniform frequency. This encoding approach not only retains essential information from the original dataset but also demonstrates resilience in downstream tasks, owing to the inherent probabilistic variability of the frequency itself.

SNNs represent an innovative fusion of principles from neuroscience and computer science, imparting a heightened level of biological fidelity in contrast to conventional artificial neural networks within the domain of AI [9]. In biological neuronal systems, information transmission occurs through electrochemical pulses, a process emulated by SNNs through the abstraction of bioelectrochemical pulses into binary states, indicative of either the presence or absence of pulses for inter-neuronal communication [16]. It must be emphasized that the biochemical processes by which living organisms convert inputs into electrochemical impulses differ significantly from existing pulse coding methods. Since biological research requires observable phenomena to validate theories, the limited capabilities of our current observation tools may constrain our exploration of encoding mechanisms in the brain [19].

The ocular apparatus is a complex system wherein cells engage in intricate processing of external stimuli before relaying information to neuronal cells. We hypothesize that this pre-processing encompasses the mechanism of spiking encoding. Specifically, we assume that spiking encoding is not merely a direct numerical conversion but rather a process that intrinsically considers the interplay between distinct spatial information. We hypothesize that the brain’s encoding mechanisms for text, sound, and other modalities may involve complex, unobserved processes. Given the inherent limitations of current observational tools—particularly their inability to detect rapid evolutionary or dynamic phase transitions—it is reasonable to explore hypothetical frameworks. This approach does not preclude empirical validation but represents a necessary pathway to transcending existing coding paradigms.

This paper initiates a preliminary exploration into a novel direction within the domain of microparticles in biological cell, where ongoing debates persist regarding the internal processing mechanisms of biological cells. Within this discourse, one perspective posits the potential involvement of quantum mechanics in information processing at the cellular level [20]. Quantum mechanics, serving as an extension of Newtonian mechanics at the microscopic scale, represents a contemporary framework for understanding the intricacies of the natural world. Based on this, we propose the hypothesis that incorporating quantum phenomena into spike encoding may offer a novel exploratory perspective, potentially bringing the coding process closer, in certain respects, to the encoding mechanisms within the brain. Although this notion remains contentious, some scholars do posit the existence of quantum phenomena in the brain [20]. Quantum effects in biology have credible evidence in certain contexts (e.g., coherence-related explanations in photosynthesis and magnetoreception are actively studied), but we do not use these phenomena as evidence for neural entanglement; we mention them only to contextualize the term ‘quantum-inspired’. However, “entanglement” in our encoding module is used as a mathematical mechanism to introduce structured stochastic dependence between encoded spike variables, not as a literal assertion of biological entanglement. Specifically, we associate the two quantum states with the presence or absence of spikes in the encoding scheme, and then introduce adjacent feature entanglement into the spiking-encoding process, allowing data at different locations to become entangled during encoding. The resulting spike train, generated through this quantum-entanglement-based encoding, is subsequently used as input to the SNN.

To construct a quantum mechanics-inspired encoding method, it is essential to first consider “entangled information bits”—specifically, the positions occupied by the input data within the information space. Although in theory, any two information bits can be entangled, this paper focuses exclusively on the entanglement relationships between adjacent positions. Given the vast number of possible positional combinations, limiting entanglement to neighboring pairs significantly reduces the complexity of implementing the encoding process (a detailed analysis will be provided in Section 3). Subsequently, the design of quantum circuits, specifically quantum algorithms, becomes necessary to execute the quantum encoding process. It is noteworthy that in the quantum realm, quantum bits inherently exist in |0〉 state or |1〉 state. Throughout the encoding calculation phase, these quantum bits can even exist in a superposition state of in |0〉 and |1〉, a phenomenon manifest in quantum circuits. Finally, employing quantum observation, we derive a spike train comprising binary states (0/1), which is subsequently utilized for the experimental validation of downstream tasks.

Based on the above discussion, the central research question of this work is whether quantum-inspired principles, specifically superposition and neighboring entanglement, can be leveraged to construct an effective spiking encoding scheme. We hypothesize that such an encoding mechanism can achieve a favorable balance between spike sparsity and information preservation compared with conventional spiking encoding methods. This hypothesis is examined through reconstruction analysis and downstream sentiment recognition tasks.

In summary, the contributions of this paper are highlighted as follows:

- Drawing inspiration from quantum superposition states and entangled states in quantum mechanics, we introduce a spiking encoding approach that collapses quantum bits in superposition states into traditional bits through observation operations. This collapse maps the quantum bits onto the presence or absence of spikes, realizing a quantum-inspired encoding mechanism.
- To implement this encoding method, we propose a detailed implementation strategy that leverages existing quantum simulation software on conventional computing devices. This approach allows us to execute specific quantum circuits and ultimately achieve our desired encoding objective.
- To quantitatively and qualitatively assess the accuracy of spiking encoding, we employed both quantitative metrics and qualitative analysis. To validate the effectiveness of our encoding method, we selected emotion recognition as a downstream task.

## 2. Related Works

### 2.1. Emotion Recognition

Emotion recognition constitutes a domain seeking to leverage computational capabilities to emulate human emotional cognition. This encompasses the identification of human emotions [21] and the discernment of emotional responses elicited by visual stimuli [22]. Positioned as a subset within the broader spectrum of affective computing, emotion recognition assumes a pivotal role in the realm of AI investigations. The precise recognition of emotions carries significant implications for diverse applications, including but not limited to autism diagnosis [23], online educational platforms [24], intelligent vehicular systems [25], and other multifaceted domains.

Human emotion recognition encompasses diverse modalities, and existing methodologies can be categorized into accuracy-oriented [12], robustness-oriented [26], and efficiency-oriented approaches [14], contingent upon specific research objectives. The accuracy-oriented approach is centered on the development of sophisticated neural networks or alternative methodologies to enhance the accuracy of emotion recognition, representing a predominant focus in current research endeavors. The robustness-oriented approach scrutinizes the resilience of emotion recognition models in the presence of challenges or interference, with the objective of identifying robust solutions. Meanwhile, the efficiency-oriented approach directs attention to energy consumption, with the aim of reducing and optimizing the computational processes inherent in model inference during emotion recognition. This strategic focus not only contributes to efficiency but also aligns with broader goals of energy conservation and environmental preservation.

This paper introduces an emotion recognition methodology based on SNN, notable for its pronounced advantage in terms of energy efficiency and environmental sustainability [27]. The primary focus of this paper centers on the intricacies of spiking encoding within emotion recognition frameworks employing SNNs. Given the multifaceted nature of this challenge and the inherent multimodal characteristics of emotion recognition data [28], we have selected this application as an illustrative context to showcase the effectiveness of the proposed spiking encoding strategy.

### 2.2. Spiking Neural Networks

The inception of SNNs was aimed at addressing pronounced deviations in biological fidelity when emulating neurons, compared to the conventional Artificial Neural Network (ANN) [16]. A prominent drawback of prevalent ANN architectures lies in their substantial energy consumption, which poses environmental concerns as a result of associated energy generation processes [29]. A foundational behavioral analysis underscores that the elevated energy consumption in ANN primarily emanates from the partitioning of computation and storage within the established von Neumann architecture during the calculation process. In this context, energy expenditure for data handling significantly surpasses that of data computation. The design principle of SNNs circumvents this challenge by operating on neuromorphic chips [9], thereby mitigating energy consumption associated with data handling. Moreover, in practical deployment scenarios, prevailing neural networks conventionally employ 32-bit precision for training calculations, acknowledging that floating-point numerical multiplication calculations incur comparatively elevated energy costs. Consequently, for optimal reasoning and deployment, it becomes imperative to quantize operations to the greatest extent feasible [30]. The operational paradigm of SNNs notably replaces multiplication with accumulation.

The transmission of information within biological neurons is governed by intricate biochemical mechanisms. Neurons propagate information through bioelectric pulses, necessitating a voltage differential created by the ion concentration gradient across the cellular membrane. An initial random pulse sequence is generated. Subsequently, the neuron’s synapse modifies the current membrane potential through its individual current. It is evident that the incoming pulse sequence induces adjustments in the current magnitude of the synapse connected to the current neuron, each modification occurring in increments of 1, albeit exhibiting a subsequent descending trend. This trend corresponds to a concomitant decline in membrane potential, particularly in the absence of external or previously connected neurons. The establishment of a membrane potential threshold prompts the emission of spikes when a specified value is exceeded, resulting in an abrupt reduction in the membrane potential. This intricate process has prompted the proposition of numerous spiking neuron models, with the Leaky Integrate-and-Fire (LIF) neuron standing as the most prevalently utilized.

**Figure 1:**
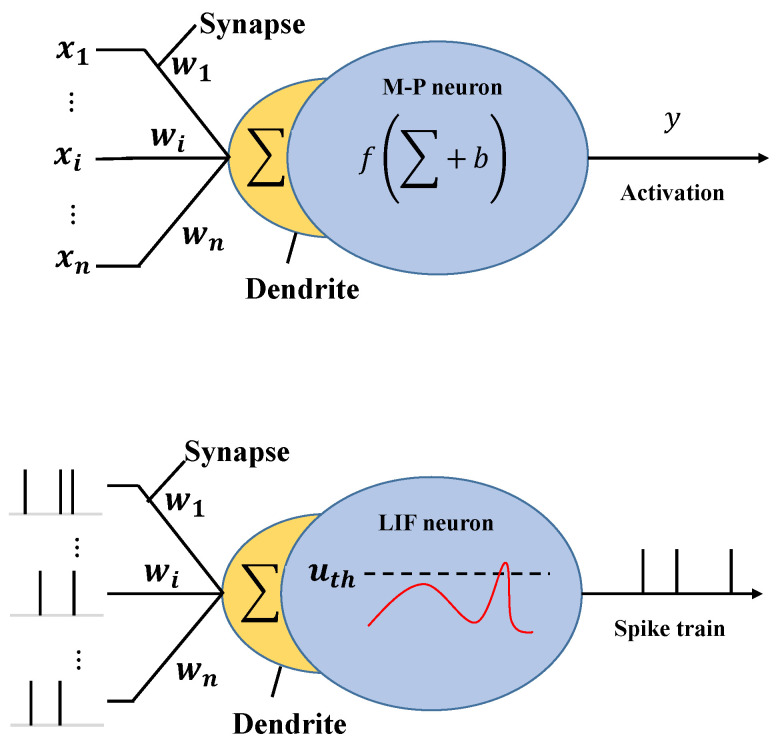
Neurons in ANN (**above**) and SNN (**bottom**).

The traditional neuron, commonly used in ANN, referred to as the McCulloch-Pitts neuron [31], adheres to a mathematical formulation involving the weighting of inputs (typically represented as floating-point values), addition of a bias, and subsequent passage through an activation function to yield the output. Conversely, the input spike train in LIF neurons within spiking neuron models undergoes a temporal weighting and summation process. However, owing to the presence or absence of spikes during input, the original multiplication operation in McCulloch-Pitts neuron undergoes transformation into an accumulation operation. Subsequently, a voltage threshold comparison operation transpires within the neuron cell. Should the membrane potential at a specific time exceed the threshold, a spike is emitted accordingly; otherwise, an exponential decay operation is applied to the membrane potential. According to [27], the energy consumption associated with a singular multiply-accumulation operation and an accumulation operation is reported as 4.6 pJ and 0.9 pJ in 45 nm 0.9 V chip, respectively. To visually illustrate the distinctions between LIF neurons [32] and conventional neurons [31], Figure 1 shows the details.

**Figure 2:**
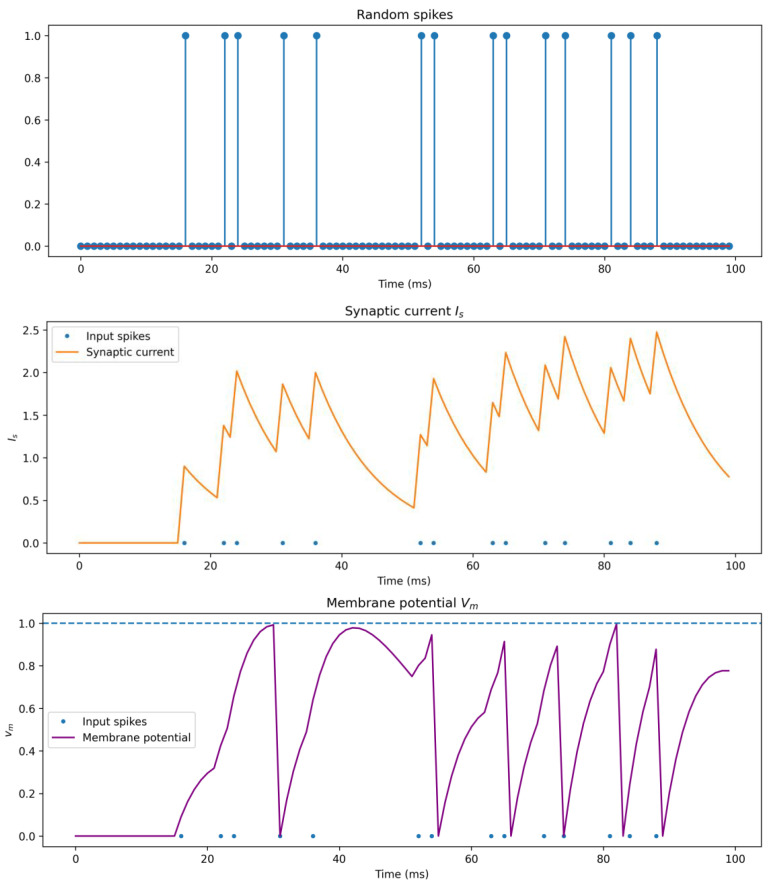
Dynamics of membrane potential in spiking neuron. The red line in the figure represents the baseline, which corresponds to the numerical value in the absence of spikes. To better distinguish between different data points, a blue dot is used to mark the data at each time step, regardless of whether the marker represents 0 or 1.

The dynamic changes of neurons in SNNs are time-dependent, which is significantly different from ANNs. As shown in the Figure 2, the top subplot represents the input spike trains, with the horizontal axis indicating time. The bottom subplot illustrates the membrane potential of the neuron. It can be observed that the membrane potential changes, typically increasing, as spikes arrive. However, once the membrane potential exceeds the set threshold potential, the membrane voltage rapidly drops to zero and begins to accumulate again on the basis of the input spikes. Regardless of the type of spiking neuron, the overall abstract logic remains the same.

### 2.3. Quantum Theory

**Figure 3:**
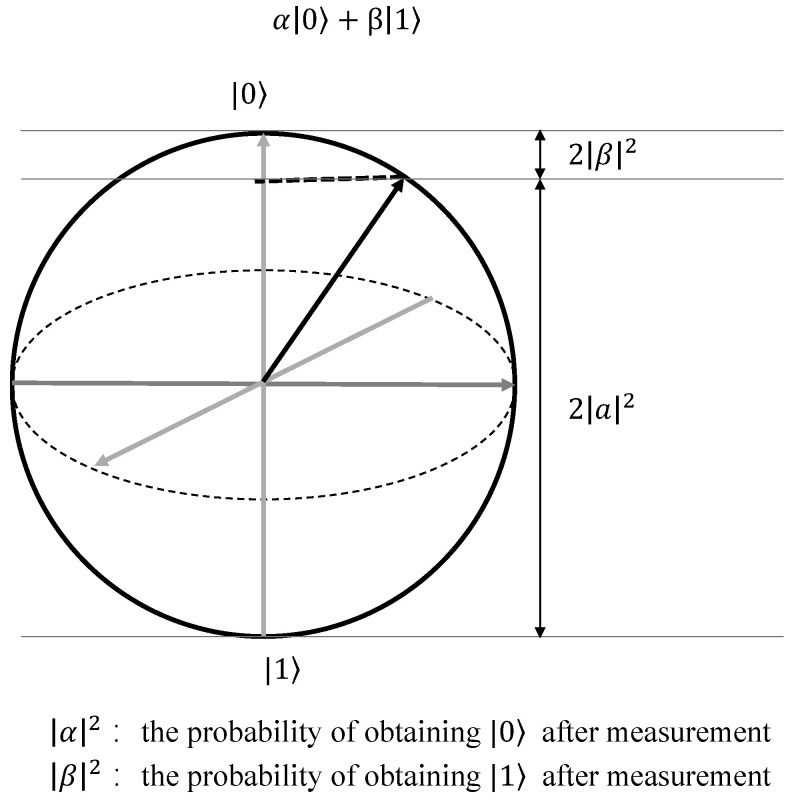
Bloch sphere: schematic diagram of quantum observation process.

While the three laws of Newtonian mechanics provide a robust framework for comprehending phenomena at the macroscopic level, their explanatory power diminishes when applied to observations at the microscopic scale. In contrast, the preeminent descriptor of mechanical changes in the microscopic realm is the Schrödinger equation [33]. Quantum theory, arising from an exploration into the composition of matter, introduces a pivotal concept: the inherent uncertainty associated with microscopic particles. Specifically, a microscopic particle, hypothetically existing in two discrete states, has the capacity to exist in a superposition state wherein both states concurrently subsist. Upon observation, this superposition state undergoes collapse, manifesting as one of the two states [34]. This process can be vividly depicted through the visualization of a Bloch sphere, as illustrated in the Figure 3.

The theoretical principles emanating from the investigation of material composition find widespread application in the realm of computer science. Within the domain of natural language processing, a pervasive challenge involves semantic contradictions and ambiguities within sentences, wherein a singular sentence may convey multiple meanings. This intricacy bears a direct connection to the concept of uncertain states in quantum theory, marking an early instance of quantum theory’s application in computer science [35]. Consequently, subsequent applications have surfaced across diverse tasks, including but not limited to information retrieval [36] and emotion recognition [37].

In the field of quantum computing and SNNs, some efforts have been made to integrate quantum theory with SNNs. Kristensen et al. [38] introduce a novel concept that combines the fields of neuromorphic computing and quantum mechanics. The primary objective is to quantize the discrete spiking model. Addressing the issue of background inversion in image processing, Sun et al. [39] suggest a method for data encoding using superposition states, they co-encode the original image and its color-inverted (complementary) counterpart into a single quantum-superposition state, then map this superposition into spike trains, thereby translating quantum information into biologically interpretable spatio-temporal spike signals. However, this approach overlooks the quantum entanglement phenomenon in quantum mechanics. Konar et al. [40] embed quantum circuits as a layer/module for classification robustness, which is conceptually different from our goal of improving the encoding that precedes SNN training/inference. Additionally, Yan et al. [41] introduce Quantum particle swarm optimization for smoothing and denoising raw data in air quality evaluation. Previous researchers provides a comprehensive review and future prospects for research on quantum computing and SNNs. Readers can refer to this paper for more detailed information [42]. Furthermore, Li et al. introduce quantum theory into multimodal information fusion for multimodal emotion recognition, thereby enhancing the interpretability of network models [43]. Ashar et al. [44] propose a Quantum-Enhanced Spiking Neural Network (QESNN) for closed-loop neuromodulation, integrating quantum sensing and neuromorphic computing to achieve highly sensitive and energy-efficient neural activity detection. Brand and Petruccione [45] introduce a novel Quantum Leaky Integrate-and-Fire neuron model implemented as a compact quantum circuit using only two rotation gates. These neurons are used to build Quantum Spiking Neural Networks and Quantum Spiking Convolutional Neural Networks. Innan et al. [46] introduce the FL-QDSNNs framework, integrating Quantum Spiking Neural Networks (QSNNs) with Federated Learning (FL) for enhanced privacy and performance in distributed learning. Ajayan and James [47] propose a hybrid image classifier using quantum circuits and SNNs. The quantum circuit serves as the classifier, leveraging the dynamic behavior of SNNs and the parallelism of quantum computing. Konar et al. [48] integrate a classical SNN with a variational quantum circuit (VQC). The SNN converts input images into spike trains, while the VQC processes these spikes and performs classification. It can be observed that the quantum-inspired process does not act as a spike encoding component in any of these methods. That is to say, it does not involve the procedure of encoding floating-point setting values into spike trains, and this is exactly the research problem addressed in this paper.

To the best of our knowledge, this paper introduces a groundbreaking integration of quantum theory into the realm of spike encoding which is omitted by existing works. Our conceptual foundation originates from recognizing the inherent uncertainty associated with the emission of bioelectric pulses from neurons in biological systems, postulating a potential correlation with the intrinsic uncertainty embedded in quantum mechanics [34]. Significantly, the binary representation (0/1) of the presence or absence of biological pulses aligns with the analogous states of quantum bits, denoted as |0〉 and |1〉 according to widely-used Dirac Notations, known as “bra-ket” notation [34], respectively. This correlation establishes a connection between the uncertainty characterizing electrical pulses in biology [20] and the uncertainty inherent in quantum bits.

## 3. Proposed Method

**Figure 4:**
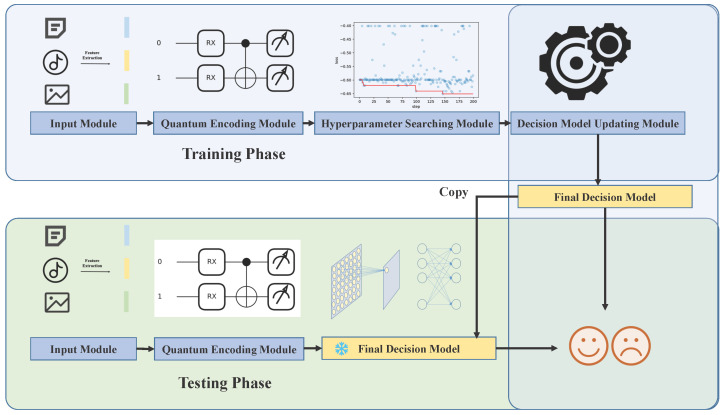
The framework of the proposed method.

The framework of the proposed method is illustrated in Figure 4, consisting of two main components: training phase and testing phase. During the training phase, compressed features are obtained through input module. Subsequently, these features undergo processing in the quantum encoding module, resulting in spike trains that are used to train the SNN. To optimize hyperparameters, the hyperparameter searching module leverages historical training parameter performance as prior knowledge. The final decision model is further refined in the decision model updating module.

During the testing phase, the first two modules operate similarly to those in the training phase. However, rather than updating the decision model, it is directly utilized to infer the emotional category output. Please note that as testing is inherent in the training process, only the training phase will be described in detail herein.

### 3.1. Input Module

The purpose of this module is to condense the raw input data into compressed features. As this paper focuses on emotion recognition based on sequential data, a set of feature vectors is generated. To maintain consistency with prior research, we employ publicly available benchmark features [49]. It is assumed that the feature data can be represented as $x_{1},x_{2},\ldots ,x_{L}$, where *L* is the length of the sequence, and the dimension of each vector is dim.

It is essential to highlight that the adoption of this step is motivated by the current stage of quantum computing development, where excessive data can impose computational burdens in simulated environments. Our primary objective is to validate the efficacy of quantum encoding of spike trains, hence extracting features does not compromise our research objectives.

Taking text as an example, the features used are derived from GloVe, a classic tool that maps words to word vectors. The selected word vectors have a feature dimension of 300, meaning each word is ultimately mapped to a vector of length 300, where each element in the vector is a floating-point value.

### 3.2. Quantum Encoding Module

The role of this module is to encode feature data into quantum states using quantum circuits, and subsequently generate corresponding spike trains of a desired length through multiple observations. To begin, let us delve into the intricacies of quantum circuits. These circuits serve as the backbone for encoding feature data. We will then explore the intricate process of encoding feature data into quantum circuits. Finally, we will outline the process of deriving spike trains through observation.

The fundamental entity manipulated within quantum circuits is the quantum bit, often denoted as a qubit. A qubit in a superposition state can be described using Dirac notation, as follows:

(1) |ψ〉=α|0〉+β|1〉

where α and β are complex numbers satisfying ${\left|\alpha \right|}^{2}+{\left|\beta \right|}^{2}=1$. Here, the squared probability amplitude represents the likelihood of the superposition state collapsing to a specific quantum state upon observation. This superposition state was leveraged in [39]. However, beyond the superposition state, another remarkable feature of quantum bits is their capacity for quantum entanglement. Quantum entanglement refers to the mutual influence between two quantum bits.

**Figure 5:**
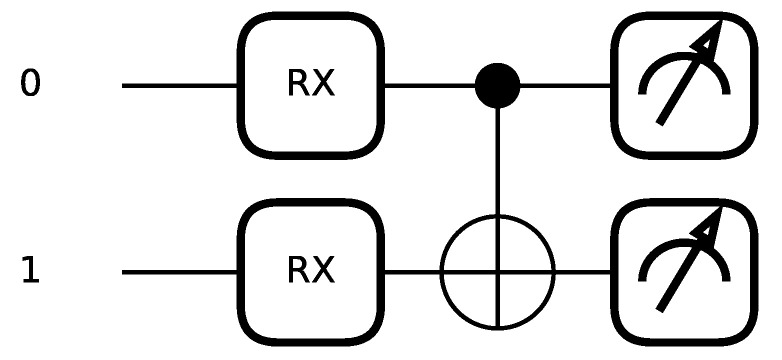
The quantum circuit used to generate spike trains.

Motivated by the principles of quantum superposition and quantum entanglement, we employ the quantum circuit depicted in Figure 5 for spike trains generation (This figure is generated by the simulation software: https://pennylane.ai/ (accessed on 25 April 2025)). This circuit entails two quantum bits that undergo sequential operations. Initially, these bits traverse through an RX (X Rotation) gate with specific parameters to achieve a superposition state. Subsequently, they encounter a CNOT (Controlled-NOT) gate to generate an entangled state. Finally, measurements are conducted on both bits to determine the ultimate collapsed result. This circuit, including the observation process, can be simulated using PennyLane v0.31.0, a software package. The code corresponding to the aforementioned encoding process is presented in Listing 1. As shown in the code, during each spike encoding step, the two selected floating-point numbers are passed as rotation angle to the RX gate. They are then entangled via a CNOT gate. Finally, the output of a single spike is obtained by measuring both qubits with the Pauli-Z observable. Repeating this process multiple times, this paper recommends selecting a value between 500 and 1000 iterations, which yields spike trains of corresponding length.

**Listing 1.** Python code of the proposed encoding.

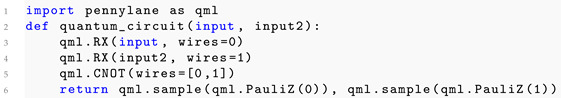



Mathematically, two qubits are initialized in the |0〉 state and independently transformed by RX gates with rotation angles given by the paired input feature values. For each qubit, the RX(θ) operation produces the state

(2) $$|\psi \left(\theta \right)\rangle =\cos\left(\frac{\theta }{2}\right)\;|0\rangle +\sin\left(\frac{\theta }{2}\right)\;|1\rangle$$

After these rotations, a CNOT gate is applied to introduce correlations between the two qubits. The final spike values are obtained by sampling the Pauli-Z observable on each qubit. Repeated sampling yields binary spike trains whose empirical distributions are determined by the rotation-induced amplitudes and their coupling through the CNOT operation.

It should be emphasized that the RX gate is intentionally used in this work as a data-dependent generalization of the Hadamard operation. While a Hadamard gate produces a fixed balanced superposition with equal probability amplitudes, the RX(θ) gate generates an input-conditioned and generally unbalanced superposition, where the amplitudes are directly determined by the feature values through the rotation angle θ. Therefore, balanced superposition is not the objective of this study. Instead, we leverage data-adaptive superposition, together with local entanglement, to construct a probabilistic spike-encoding mechanism that reflects input-dependent uncertainty.

**Figure 6:**
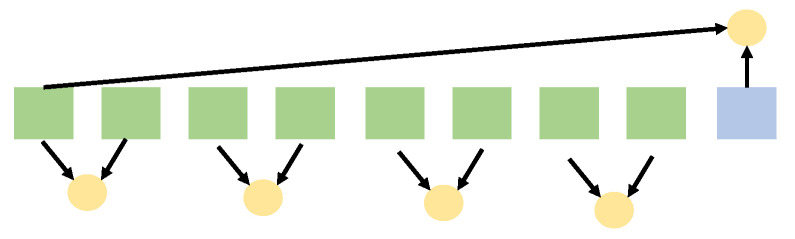
Spatial selection for spiking encoding: the square means the pixel and the circle denotes the entanglement.

Despite the potential for various spatial configurations in reality, for simplicity’s sake, we primarily consider the entanglement relationship between adjacent feature values within the feature vector. The simplification is indeed possible to lead to potential information loss restricting entanglement edges can miss long-range correlations. Note that the adjacency pattern is a baseline choice rather than theoretically optimal. To clarify, for any given vector, we apply the aforementioned quantum circuit operations to its adjacent values. As shown in Figure 6, since the length of the vector is not necessarily even, our proposed method of considering two spatial positions for entanglement and superposition may miss information in cases where the length is odd. To compensate for this loss, in the case of odd lengths, we fuse the information of the last number with the first number. It should be noted that this is only one concrete implementation; our goal is to verify the feasibility of the encoding itself, and other alternatives—such as leaving the final feature uncoupled, padding a dummy feature, or employing a learned/sparse pairing—are equally viable. Ultimately, we obtain the spike trains by observing the underlying measurement basis. Given that the input feature sequence can be represented as a matrix $X_{L\times dim}$, the sampled spike trains can be formulated as $X_{L\times dim\times T}$, where the *T* represents the sample times of the output qubits of quantum circuit.

## 4. Formalized Processing Flow

We aim to generate a binary pulse sequence $P=(p_{1},p_{2},\ldots ,p_{L})$ of length *L* from the input features $\left(x_{1},\ldots ,x_{L}\right)$.

### 4.1. Case 1: *L* Is Even

- Pair the features sequentially into $\frac{L}{2}$ groups:
  $$\left(x_{1},x_{2}\right),\;\left(x_{3},x_{4}\right),\;\ldots ,\;\left(x_{L-1},x_{L}\right)$$
- For each pair $\left(x_{2k-1},x_{2k}\right)$, feed it as rotation angles into the quantum circuit *Q* as shown in Listing 1 and obtain the corresponding two output pulses:
  $$\left(p_{2k-1},\;p_{2k}\right)=Q\left(x_{2k-1},x_{2k}\right),\;k=1,\ldots ,\frac{L}{2}$$

### 4.2. Case 2: *L* Is Odd

1. Process the first L−1 features in pairs:
  - Form $\frac{L-1}{2}$ groups:
    $$\left(x_{1},x_{2}\right),\;\left(x_{3},x_{4}\right),\;\ldots ,\;\left(x_{L-2},x_{L-1}\right)$$
  - For each group, apply Q:
    $$\left(p_{2k-1},\;p_{2k}\right)=Q\left(x_{2k-1},x_{2k}\right),\;k=1,\ldots ,\frac{L-1}{2}$$
2. Process the last feature $x_{L}$:
  - Pair $x_{L}$ with the first feature $x_{1}$ and input $\left(x_{1},x_{L}\right)$ into Q:
    $$\left({\mathrm{temp}}_{1},\;{\mathrm{temp}}_{L}\right)=Q\left(x_{1},x_{L}\right)$$
  - Since $p_{1}$ is already determined in step 1, we only retain the output corresponding to $x_{L}$ (as stated: “*only observe the output corresponding to $x_{L}$*”):
    $$p_{L}={\mathrm{temp}}_{L}$$
  - The other output ${\mathrm{temp}}_{1}$ is discarded.

Assuming the feature dimension is 300, and given that the designed encoding circuit takes dual inputs—meaning only entanglement between two bits is considered—if we were to account for all possible entanglement pairs, we would need to select any two of the 300 values for encoding. This would result in a total of 44,850 possible cases. While such an approach would yield a more comprehensive coverage of entanglement scenarios, it would impose a significant computational burden on the simulation hardware, such as PennyLane. To mitigate simulation complexity, a trade-off is made in this work: only adjacent feature values are considered for entanglement. Specifically, entanglement is applied between each pair of adjacent features, which reduces the encoded feature dimension to 300. If the number of features is odd, the remaining single feature is entangled with the first feature to prevent information loss. We emphasize that the proposed circuit is quantum-inspired and aims to provide a structured probabilistic encoding mechanism, rather than a strict physical realization of a quantum system.

### 4.3. Hyperparameter Searching Module

**Figure 7:**
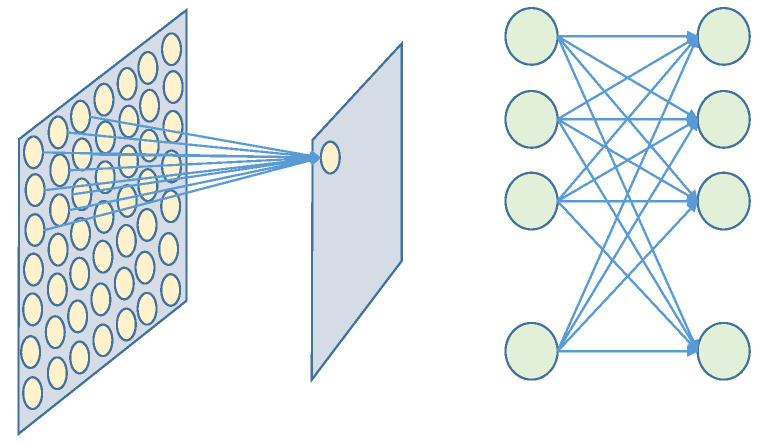
Localized connectivity and global connectivity: convolutional operations and fully connected operations.

The purpose of this module is to pick up the optimal the hyperparameters of the model. As the focus of this paper lies in spike encoding methods, we do not delve into custom designs for SNNs. The connection patterns in neural network topology represent the ways in which different neurons are interconnected. After dividing a neural network into layers, the inter-layer connection patterns can generally be categorized into global connectivity and localized connectivity. Instead, we adopt an existing model [14], using LIF neurons as our spiking neurons. The topological connections encompass convolution, pooling, and full connection, as shown in Figure 7. In addition to convolutional and fully connected layers that represent connections, the pooling layer is a technique that can downsample feature maps in a parameter-free manner and is also used in this paper to compress the output information.

Given that LIF possesses inherent voltage thresholds and time constants, we consider both parameters as candidates for hyperparameter selection. Furthermore, a parameter (volume factor) that adjusts the number of neurons is integrated into the network architecture, enabling joint optimization of the learning rate. The output of the final pooling layer represents the average firing rate of spiking neurons in output layer, indicating the likelihood of assigning a particular category. We employ softmax as our classification loss function to train the network with surrogate gradient.

We employ a Bayesian-based hyperparameter optimization algorithm [50]. The characteristic of this optimization process lies in the recording of hyperparameter combinations and their corresponding performance metrics from previous searches. Subsequently, a distribution is constructed to approximate this process. In subsequent parameter selection efforts, this distribution serves as a prior, mitigating the arbitrariness of random parameter combinations.

### 4.4. Decision Model Updating Module

After determining the optimal hyperparameters, we increase the number of training epochs from 20 to 100 in this module to obtain the final decision model. Upon obtaining the final decision model, we can analyze test results by feeding test data into the trained model to obtain our final inference results.

## 5. Experiments

### 5.1. Datasets

Given the current computational cost of quantum-circuit simulation, using benchmark features is a pragmatic choice to validate the encoding mechanism itself without conflating results with a learned frontend. The Multimodal Opinion Sentiment Intensity (CMU-MOSI) corpus, introduced in [28], represents a widely recognized benchmark in multimodal sentiment analysis research. This dataset comprises 2199 carefully extracted video clips sourced from 93 distinct movie review videos on YouTube. It is systematically partitioned into three subsets: 1284 segments for training, 229 for validation, and 686 for testing. Every video segment is accompanied by a fine-grained sentiment intensity label, which is assigned a continuous numerical value between −3 and +3, reflecting the polarity and strength of the expressed opinion.

Compiled by Busso and colleagues in 2008 [51] at the Signal Analysis and Interpretation Laboratory (SAIL), the Interactive Emotional Dyadic Motion Capture (IEMOCAP) dataset is a multi-modal resource featuring interactions from multiple speakers. It provides a rich composite of emotional expression data, integrating synchronized video, audio recordings, facial motion capture information, and corresponding textual transcripts. To maintain consistency with downstream experimental settings, we selected the standard class “Happy” and constructed a binary classification task around it. The corpus is partitioned for machine learning applications into dedicated training, validation, and testing subsets, containing 2717, 798, and 938 samples respectively.

Feature extraction from the raw video segments is conducted by processing three separate data streams, following conventional practices in the field. The specific procedures for each modality are outlined below. For textual data, a pre-trained word embedding model [52] is employed to transform words into continuous vector representations. Visual features are obtained by applying the Facet toolkit to derive frame-level representations from the video. Acoustic representations are generated from the audio tracks using the COVAREP framework [53]. To achieve word-level synchronization across these modalities, we utilize the Penn Phonetics Lab Forced Aligner (P2FA) [54], a forced alignment system based on Gaussian mixture models. The final dimensionalities of the extracted features are: for CMU-MOSI, text (300), visual (20), and acoustic (5); for IEMOCAP, text (300), visual (35), and acoustic (74).

### 5.2. Experimental Setup

To accommodate distinct features, we modified the initial layer of the model, replacing the original convolution technique tailored for raw visual data with a more suitable one-dimensional convolution tailored for features. Additionally, to minimize parameter optimization complexity, the membrane voltage threshold and time constant for LIF neurons in the same layer are shared. The Hyperopt library is used to carry out hyperparameter search, and the concrete parameter types and ranges are listed in the Table 1. All ranges were chosen by slightly expanding the default values recommended by the SpikingJelly community. In a pilot study, we ran 200 Hyperopt trials and observed that the search had essentially converged after 50 evaluations. To shorten the turnaround time of the tuning loop, we fixed the number of training epochs in each trial to 20. Specifically, the membrane voltage threshold is between 1 $\times \;10^{-3}$ and 2, time constant is between 1 + 1 $\times \;10^{-3}$ and 5, the learning rate is between 1 $\times \;10^{-3}$ and 5 $\times \;10^{-1}$, the volume factor is between 4 and 12 with type integer, and the coefficient of L1 norm is between 0.01 and 2. We set a fixed random seed of 3407 for all experimental runs on CMU-MOSI and IEMOCAP.

We compared two prevalent spiking encoding techniques: rate encoding and temporal encoding, which are traditional spiking encoding approaches. To conduct our analysis, we utilized SNN and employed a widely utilized framework within the community to implement these two widely used encoding methods [55].

### 5.3. Results

Firstly, we conducted experiments on natural language processing data. As spiking encoding length is a crucial parameter in spiking encoding, we tested multiple lengths, varying from 100 to 1000 with a 100 interval. The results for the language modality are summarized in the ‘L’ part of Table 2. Specifically, ‘R’ represents the rate encoding, ‘T’ represents temporal encoding and ‘Q’ represents the proposed quantum encoding. As the encoding length T increases, there is an overall upward trend in emotion recognition accuracy. However, it is noteworthy that certain dimensions exhibit fluctuations in accuracy. Specifically, for rate encoding, there were aberrant variations at 500 and 1000 that deviated from the overall trend. Our pipeline uses Bayesian hyperparameter optimization, so different T values may end up with different “best-found” settings under a finite search budget; a suboptimal selection at a specific T can create an apparent dip even if the underlying trend is monotonic. One potential solution is to mitigate these variations by averaging results from multiple independent experiments. However, considering the involvement of hyperparameter search throughout the computational process, the average computational cost associated with multiple experiments becomes relatively high. The primary objective of this study is to validate the efficacy of the encoding method. We suggest that occasional fluctuations in the data will not undermine the evidence supporting the encoding’s effectiveness.

Secondly, we conducted experiments on audio data, and the outcomes are detailed in the ‘A’ part of Table 2. In downstream tasks, the performance of rate encoding and quantum encoding falls short of temporal encoding, a finding that contradicts common sense. Our detailed analysis reveals that this outcome is attributed to the sparsity of temporal encoding combined with the limited dimensionality of audio features, specifically the length of audio feature vectors being 5. Consequently, less effective information is transmitted, leading to an unexpected situation where all output results are zero. While this scenario is theoretically flawed, it is nevertheless recognized as a valid judgment by the code’s logical evaluation, resulting in the erroneous perception of the model’s performance as excellent. In comparing rate encoding with enquantum coding, the latter demonstrates advantages in dimensions 400, 600, 700, and 800. However, it is important to note that these advantages also encompass the aforementioned failure scenario. It should be emphasized that “effectiveness” is always tied to a specific encoding on a specific dataset for a specific task. We therefore use downstream-task evaluation merely to explore when an encoding works, not to claim universal superiority. When the underlying unimodal features provide limited discriminative information for the target labels, downstream performance will remain near the corresponding majority baseline regardless of the encoding. For example, on CMU-MOSI, both audio and visual features frequently yield emotion-classification accuracies with SNNs that are below random guessing, regardless of the encoding scheme employed. Therefore, the assessment of downstream tasks serves as a mere reference, and a more insightful comparison of coding methods can be found in the Section 5.4.

Finally, we conducted experiments on visual data, maintaining similar experimental conditions as those used for natural language. The results for the visual modalities are presented in the ‘V’ part of Table 2. It is evident from these results that the proposed quantum encoding’s overall accuracy falls between rate encoding and temporal encoding in terms of performance. While acknowledging that the model’s accuracy has lost its practical relevance for downstream tasks, given that emotion classification accuracy often falls below the 50% threshold of random guessing, we still observe a consistent trend. Specifically, rate encoding maintains the highest overall accuracy. When compared to temporal encoding, quantum encoding demonstrates a relatively higher overall accuracy. Therefore, this experiment underscores the advantages of quantum encoding over temporal encoding, even when considering encoding alone, despite its limited practical significance.

### 5.4. Analysis

In addition to the aforementioned data-driven analysis of downstream tasks, we also conduct encoding performance evaluations from the perspectives of reconstruction visualization and reconstruction quantization errors. To reconstruct the visual representation, we employ a spike counting approach based on the encoded data, followed by the visualization of the feature data. The reconstruction quantization error is quantitatively assessed using the Root Mean Square Error (RMSE). We calculated the RMSE for all data associated with each modality. As shown in Table 3, the minimum RMSE achieved by rate encoding indicates its closest approximation to the original data. In contrast, the highest RMSE observed for temporal encoding is primarily attributed to its inherent sparsity. Quantum encoding exhibits an RMSE that lies between the two, representing a balanced trade-off between the two encoding methods.

**Figure 8:**
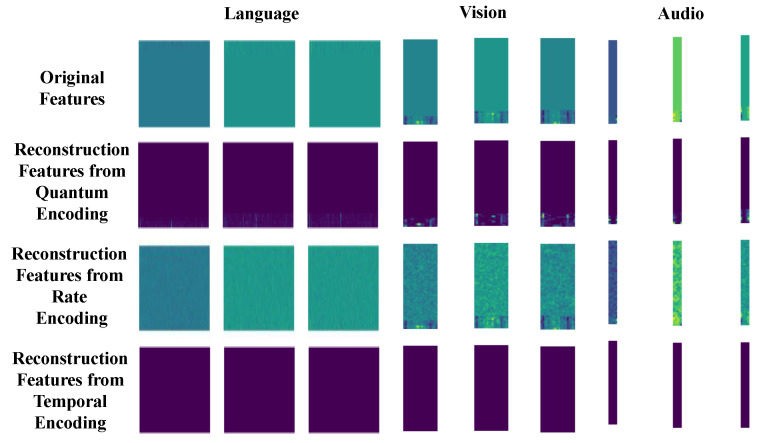
Visual examples of original features and reconstruction of different spiking encoding methods.

For simplicity, we selected three datasets for each modality and compared the visual reconstruction errors of our proposed encoding method, rate encoding, and temporal encoding. Figure 8 reveals that rate encoding offers the closest approximation to the original image. Conversely, quantum encoding and temporal encoding exhibit greater similarity. According to the quantization table, quantum coding serves as a balance between high sparsity and minimal reconstruction error, highlighting its advantages.

**Figure 9:**
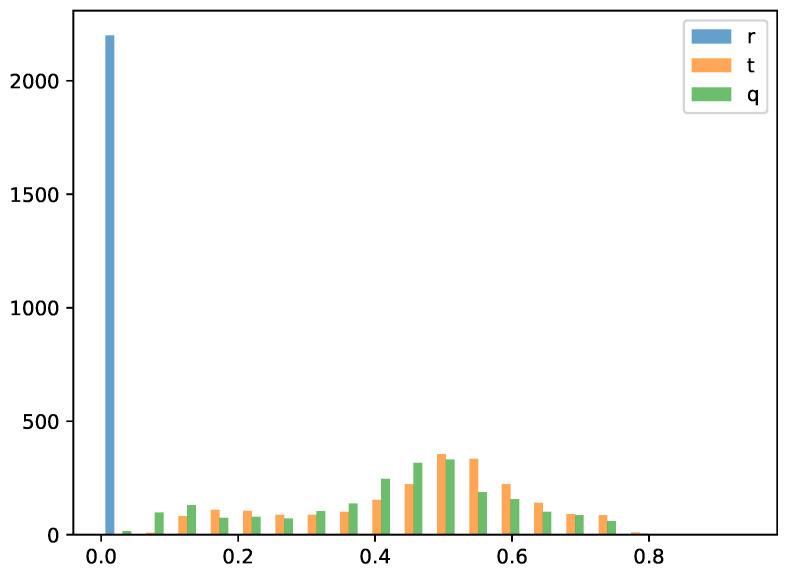
Histogram of RMSE Distribution for Different Encoding Methods.

To statistically verify the effectiveness of the proposed encoding method, we plotted the histogram of RMSE distribution. As can be seen from the Figure 9, the distribution of quantum encoding is more similar to that of temporal encoding, while most of the RMSE values of rate encoding are close to zero. This is because rate encoding is numerically closest to the original values, whereas temporal encoding and quantum encoding provide a time or entanglement based encoding approach. However, during the calculation process, the compression of the time dimension within the simulation cycle directly leads to the loss of relevant temporal information, with only the information about the number of spikes retained. This indicates that rate encoding is insensitive to the spike firing time. On the contrary, both temporal encoding and quantum encoding are more sensitive to the spike firing time.

Therefore, an intuitive way to verify the effectiveness of the encoding methods still lies in the performance of downstream tasks. The Table 2 presents the experimental results of different encoding methods at various time steps, which are equivalent to multiple tests on the encoding methods. We conducted paired *t*-tests, and the results are shown in Table 4, where L, A, and V represent the linguistic, audio, and visual modalities respectively, while Q, R, and T denote quantum encoding, rate encoding, and temporal encoding. The alternative hypothesis we selected was set as greater. For instance, the row of LQ and LR represents the statistical result where the alternative hypothesis was defined as the performance of LQ being greater than that of LR. Since the calculated *p*-value was 0.85, which is greater than the significance level of 0.05, we fail to reject the null hypothesis. In other words, the data do not provide evidence that LQ performs better than LR. This finding is consistent with the results of our previous intuitive numerical comparison. An analysis of the statistical results reveals that the cases where the *p*-value is less than 0.05 only occur in the linguistic modality (L), where Q and R are significantly better than T. The same pattern also applies to the visual modality (V).

To account for the anomalous results observed in the audio modality, we attribute this phenomenon to the insufficiently low dimensionality (merely 5 dimensions) of the original audio feature vectors. Consequently, we adopted the IEMOCAP dataset, which provides audio feature vectors with a dimensionality of 74, and the corresponding results of emotion recognition are presented in the Table 5. Herein, Qwoe denotes the quantum encoding method without entanglement, while Qrande represents the variant where instead of employing the original neighbor-based feature processing approach proposed in this paper, two features are randomly selected for encoding. It can be observed that for the audio modality, the quantum encoding method with entanglement yields superior downstream performance (86.0% vs. 85.9%). Notably, even when entanglement is implemented with random feature pairing, a performance improvement can still be achieved.

A comprehensive comparison of Table 2 and Table 5 reveals that the effectiveness of encoding methods for the audio modality is correlated with the dimensionality of features: for audio feature vectors of varying lengths, the performance of different encoding methods varies significantly. Specifically, for features with inherent weak discriminability, probability-based encoding methods may not be an optimal choice. Only when the features themselves possess strong class discriminability can rate encoding or quantum encoding yield superior performance. Within the affective computing community, publicly available features are generally adopted directly for experimental comparisons to ensure fairness. It should be noted that although our method is not comparable to other approaches in terms of classification accuracy, it is designed to handle different modalities separately, which still demonstrates its effectiveness.

**Figure 10:**
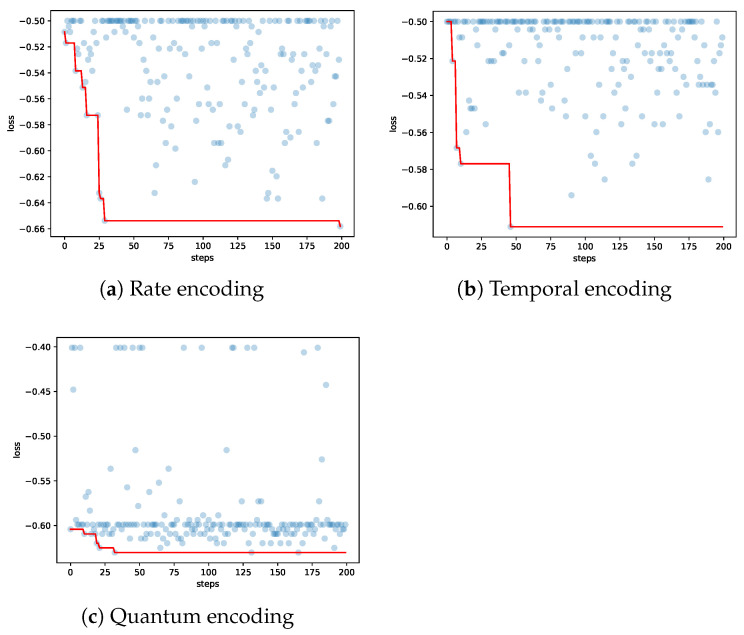
The hyperopt search process and corresponding loss. The blue dots denote the objective value of each trial. The red line tracks the best performance observed up to and including the current trial.

Figure 10 depicts the Bayesian hyperparameter search procedures corresponding to different encoding methods. Evidently, the model achieves a significant performance boost on downstream tasks during the first 50 search trials. In addition, the entire search process presents non-negligible fluctuations, which further explains the counterintuitive result in Table 2 that the classification accuracy of rate encoding when length of spike train is 1000 is inferior to that obtained when length is 500.

### 5.5. Encoding Complexity Analysis

Our encoding applies a fixed 2-qubit circuit to adjacent feature pairs and repeats measurements to generate spike trains (shots). Given an input feature sequence $X_{L\times dim}$, the sampled spike tensor is $X_{L\times dim\times T}$, where *T* is the number of circuit observations. Specicficaclly, Number of paired circuits per time is approximately dim/2. Total circuit executions across a sequence are therefore on the order of: #shots≈L·(dim/2)·T. The dominant growth with data size is linear in *L*, dim, and *T*: Time=O(L·dim·T) (with a constant factor from 2-qubit simulation). Memory to store the spike tensor also scales as O(L·dim·T), consistent with producing $X_{L\times dim\times T}$.

Classical rate/temporal encoders typically generate *T* spike samples per feature element as well, so they share the same big-O dependence on *L*, dim, *T*, while our method may incur a higher constant factor due to quantum-circuit simulation overhead. It should be emphasized that the complexity discussed here corresponds to the concrete implementation adopted in this paper—i.e., entanglement is restricted to adjacent elements only. As previously analyzed, entangling every possible pair of positions would cause the number of combinations to grow as a quadratic polynomial, i.e., dim∗(dim−1)/2.

## 6. Conclusions

We delve into the spiking encoding challenge within SNN. A spiking encoding approach that leverages quantum circuits is proposed, hypothesizing that the uncertainty inherent in biological pulse coding is linked to the microscopic quantum mechanical phenomena within organisms. To account for the phenomenon of quantum entanglement in quantum mechanics, we devised a spiking encoding method that capitalizes on adjacent spatial quantum entanglement. We conducted experimental validations on datasets containing three distinct modalities, thereby establishing the efficacy of this approach.

The experiments in this paper preliminarily demonstrate that non-neighbor entanglement encoding in the feature space is also effective. However, the impact of other topological structures on encoding performance remains unexplored. Given that the primary focus of this paper is to verify the validity of the entanglement concept in quantum encoding, in-depth research on the effectiveness of specific topological structures will be reserved for future work. Furthermore, we will consider cross-encoding features of different modalities, extending the step of information fusion from the network layer to the encoding layer.

## Figures and Tables

**Table 1:** Hyperparameter search space and ranges used by Hyperopt.

	Range	Type
Time constant	[1 + 1 $\times \;10^{-3}$, 5)	Float
Voltage threshold	[1 $\times \;10^{-3}$, 2)	Float
Learning rate	[1 $\times \;10^{-3}$, 5 $\times \;10^{-1}$)	Float
Volume factor	[4, 12)	Integer
Coefficient of L1	[0.01, 2)	Float

**Table 2:** The emotion recognition performance of different encoding methods on CMU_MOSI.

Length		100	200	300	400	500	600	700	800	900	1000
	R	74.6	65.0	67.8	69.7	55.7	71.1	55.2	60.2	77.3	40.4
L	T	42.9	40.5	42.3	40.5	44.3	40.4	42.1	40.4	46.6	48.3
	Q	46.4	56.4	65.6	53.8	61.9	66.1	62.0	64.4	59.7	53.4
	R	41.0	40.5	45.0	58.7	42.0	43.7	38.8	44.2	42.9	44.3
A	T	52.5	59.6	57.1	54.8	49.0	50.9	55.8	53.6	56.9	54.8
	Q	40.3	43.4	43.2	46.0	42.8	44.1	41.2	48.1	41.5	44.1
	R	48.7	46.2	52.0	42.9	45.8	40.8	47.4	42.9	51.6	50.0
V	T	40.4	44.0	42.1	40.4	47.7	40.4	48.1	40.4	44.0	40.4
	Q	46.4	43.4	40.8	47.0	49.2	44.1	57.8	49.2	42.7	50.8

**Table 3:** RMSE of different methods on different data.

Method	Language	Vision	Audio
Rate Encoding	0.0396	0.0383	0.0356
Temporal Encoding	0.4947	0.5011	0.4468
Quantum Encoding	0.4877	0.4902	0.4120

**Table 4:** Paired *t*-test for different encoding methods.

	*t*	*p*-Value	95% CI	Cohen-d	BF-10	Power
LQ, LR	−1.16	0.86	[−12.24, inf]	0.52	0.947	0
LQ, LT	6.72	0	[11.73, inf]	3.28	643.704	1
LR, LT	5.3	0	[13.66, inf]	2.58	148.169	1
AQ, AR	−0.44	0.66	[−3.33, inf]	0.15	0.67	0.02
AQ, AT	−8.69	1	[−13.36, inf]	4	0	0
AR, AT	−5.17	1	[−14.07, inf]	2.33	0.008	0
VQ, VR	0.15	0.44	[−3.58, inf]	0.07	0.623	0.08
VQ, VT	3	0.01	[1.69, inf]	1.06	8.952	0.92
VR, VT	2.9	0.01	[1.48, inf]	1.17	7.866	0.96

**Table 5:** The emotion recognition performance of different encoding methods on IEMOCAP.

	R	T	Q	Qwoe [39]	Qrande
A	86.4	85.9	86.0	85.9	86.1

## Data Availability

The original contributions presented in the study are included in the article, further inquiries can be directed to the corresponding author.
